# Myeloid-Derived Suppressor Cells and Mesenchymal Stem/Stromal Cells in Myeloid Malignancies

**DOI:** 10.3390/jcm10132788

**Published:** 2021-06-24

**Authors:** Suncica Kapor, Juan F. Santibanez

**Affiliations:** 1 Clinical Hospital Center “Dr Dragisa Misovic-Dedinje”, Department of Hematology, University of Belgrade, 11000 Belgrade, Serbia; 2 Molecular Oncology Group, Institute for Medical Research, University of Belgrade, 11000 Belgrade, Serbia; jfsantibanez@imi.bg.ac.rs; 3 Centro Integrativo de Biología y Química Aplicada (CIBQA), Universidad Bernardo O’Higgins, 8370993 Santiago, Chile

**Keywords:** myeloid malignancies/neoplasm, myeloid-derived suppressor cells, mesenchymal stem/stromal cells, T-cell immunosuppression, immunotherapy

## Abstract

Myeloid malignancies arise from an altered hematopoietic stem cell and mainly comprise acute myeloid leukemia, myelodysplastic syndromes, myeloproliferative malignancies, and chronic myelomonocytic leukemia. Myeloid neoplastic leukemic cells may influence the growth and differentiation of other hematopoietic cell lineages in peripheral blood and bone marrow. Myeloid-derived suppressor cells (MDSCs) and mesenchymal stromal cells (MSCs) display immunoregulatory properties by controlling the innate and adaptive immune systems that may induce a tolerant and supportive microenvironment for neoplasm development. This review analyzes the main features of MDSCs and MSCs in myeloid malignancies. The number of MDSCs is elevated in myeloid malignancies exhibiting high immunosuppressive capacities, whereas MSCs, in addition to their immunosuppression contribution, regulate myeloid leukemia cell proliferation, apoptosis, and chemotherapy resistance. Moreover, MSCs may promote MDSC expansion, which may mutually contribute to the creation of an immuno-tolerant neoplasm microenvironment. Understanding the implication of MDSCs and MSCs in myeloid malignancies may favor their potential use in immunotherapeutic strategies.

## 1. Introduction

Hematopoietic stem cells (HSCs) are bone marrow (BM)-resident primitive multipotent stem cells that continuously regenerate the blood system during the life of an organism [1]. Through cell-intrinsic HSC characteristics and extrinsic signals, the BM microenvironment finely controls blood production, and BM niche perturbations contribute to the emergence of hematopoietic malignancies. The BM niche is a complex structure encompassed by different cell types, such as multipotent mesenchymal stem cells (MSCs) and their progeny, a complex vascular network, nerve fibers, mature blood cells, immune cells, and several adhesion factors, growth factors, and chemokines, to regulate cell self-renewal and differentiation [2].

Myeloid neoplasms are heterogeneous malignancies that result from an uncontrolled proliferation of immature cells. The pathogenesis of myeloid malignancies implies intrinsic genetic and epigenetic alterations within the neoplastic population and a dysfunctional BM stroma that may promote the neoplastic process. The molecular and cellular interaction within the BM niche may contribute to the emergence of myeloid malignancies by modulating mechanisms related to proliferation, survival, and immune evasion [3,4]. Recently, myeloid-derived suppressor cells (MDSCs) have been involved in myeloid malignancies. Patients with myeloid malignancies display increased numbers of MDSCs in peripheral blood (PB) and BM and, due to their immunomodulatory function, may contribute to the escape of neoplastic cells from immunosurveillance, which appears to play a crucial role in maintaining the immune suppression within the tumor niche [5,6,7].

MSCs have been identified as one of the main cellular components of the BM microenvironment, with an essential role in normal hematopoiesis [8,9]. MSCs contribute to the progression of myeloid malignancies by establishing a favorable tumor microenvironment and displaying immunomodulatory properties by inhibiting the proliferation and function of immune cells, which contribute significantly to the pathogenesis of myeloid malignancies [10].

This review emphasizes the current knowledge of MDSCs and MSCs, and their active involvement in the pathogenesis of human myeloid malignancies, namely, acute myeloid leukemia (AML), myelodysplastic syndromes (MDSs), and myeloproliferative neoplasms (MPNs), including chronic myeloid leukemia (CML). Moreover, this review provides perspectives on new strategies being used to improve current therapies, which may ameliorate cancer management strategies to improve the life expectancy of patients diagnosed with myeloid malignancies.

## 2. Myeloid-Derived Suppressor Cells

It has been established that cancer progression is commonly associated with an increased number of immature myeloid cells at various stages of differentiation in the spleen and peripheral blood, and within the tumor stroma. Currently, these cells are recognized as MDSCs and are a hallmark of cancer and a central mechanism of immune evasion [5,11]. MDSCs create an immunosuppressive tumor microenvironment (TME) and are associated with poor prognosis and tumor burden [12,13]. In healthy conditions, a low frequency of MDSCs in the bone marrow is observed, and they, as non-polarized cells, maintain a basal suppressive environment. MDSCs may migrate to the periphery and differentiate into mature macrophages, dendritic cells (DCs), and neutrophils while losing their suppressive phenotype and supporting normal immune functions [14,15].

MDSCs comprise a small group of myeloid progenitors and immature mononuclear cells, such as monocytes (M-MDSCs), and are identified as cluster of differentiation (CD)11b^+^, CD33^+^, CD14^+^, CD15^−^, and human leukocyte antigen (HLA)-DR^low^, which are distinguished from HLA-DR^hi^ monocytes, whereas polymorphonuclear (PMN)-MDSCs (previously named granulocytic MDSCs) are defined as CD11b^+^, CD33^+^, CD15^+^ or CD66b^+,^ and CD14^−^ [16,17]. MDSC mouse counterparts are immune classified as CD11b^+^Ly6G^−^Ly6C^high^ for M-MDSCs, and PMN-MDSCs are defined as CD11b^+^Ly6G^+^Ly6C^low^ [18]. In addition, an early-stage MDSC (eMDSC) subtype has been described as the cells that lack the expression of either CD14 or CD15, and their specific roles remain to be defined [17].

The ability of MDSCs to support tumor growth and metastases can be broadly defined in the following functions: protection of tumor cells from immune surveillance; remodeling the tumor microenvironment; establishment of a pre-metastatic niche; and interaction with tumor cells to induce “stemness” and facilitate the epithelial-to-mesenchymal transition (EMT) [16]. MDSCs are implicated in several facets of immune regulation in diseases that involve chronic inflammation, including cancer. Several studies have reported the immunosuppressive effects of MDSCs in hepatocellular carcinoma, melanoma, prostate cancer, bladder cancer, head and neck squamous cell carcinoma and non-small cell lung cancer, breast cancer, gastric cancer, colorectal cancer, and others, thus evidencing their clinical significance [17,19,20,21].

## 3. Mesenchymal Stromal/Stem Cells

MSCs are a promising source for cell therapy and regenerative medicine. The therapeutic properties of MSCs are related to their potential for trans-differentiation, immunomodulation, and trophic factor secretion [22,23]. MSCs are functionally defined by their clonogenic potential (colony-forming unit-fibroblasts, CFU-Fs) and ability to assemble a functional BM niche *in vivo* [24]. The minimal criteria for human MSCs were defined by the International Society for Cellular Therapy (ISCT), in 2006, as follows: MSCs must be plastic-adherent when maintained under standard culture conditions; more than 95% of cells in a given population of MSCs should express CD90, CD73, and CD105 and lack the expression (less than 2% positivity) of CD45, CD34, CD14 or CD11b, CD79α, or CD19, and HLA class II surface molecules; and MSCs must differentiate into osteoblasts, adipocytes, and chondroblasts under standard conditions *in vitro* [25,26]. Furthermore, MSCs also express narrow levels of costimulatory molecules, such as CD40, CD80, and CD86 [27].

BM-derived MSCs (BM-MSCs) are also proposed to be located in the lineage-negative (lin)^−^CD45^−^CD271^+^CD140a^low/−^ fraction and can be further classified according to their CD146 expression; in this case, CD146^+^ MSC are reported to be located close to the vasculature and express hematopoietic stem cell (HSC)-supportive genes, such as insulin like growth factor 2 (IGF2), Wnt Family Member 3A (WNT3A), jagged canonical notch ligand 1 (JAG1), C-X-C motif chemokine ligand 12 (CXCL12), KIT ligand (KITLG), and angiopoietin-like 1 (ANGPTL1) [24,28,29,30]. In comparison, nestin-positive MSCs enhance the long-term multilineage reconstitution activity of human hematopoietic stem/progenitor cells (HSPCs) [31].

In cancer, in addition to the regenerative potential of MSCs, when they are incorporated within tumor stroma, they contribute to tumorigenesis, including its transition to tumor-associated fibroblasts; suppression of the immune response; promotion of angiogenesis; stimulation of EMT through the contribution to the TME; inhibition of tumor cell apoptosis; and, in general, promotion of tumor growth and metastasis [32].

BM is the most extensively studied source of MSCs, and although an invasive surgical procedure is needed for the recovery of BM-MSCs, a relatively low cell yield (0.001–0.01%) is obtained, which is inversely correlated with the age of the donor [33]. It has been described that BM-MSCs show a strong tropism towards injured tissues because the intravenous delivery of BM-MSCs results in their migration to specific sites of injury. Moreover, endogenous BM-MSCs are mobilized in response to inflammation or injury, thus increasing their numbers in the bloodstream and targeting specific tissues via active mechanisms. BM-MSCs can home in on and engraft to different types of solid tumors, such as breast, lung, pancreatic, colon, and prostate carcinomas, among other primary and metastatic tumors [33,34].

Due to their tropism to inflammatory sites, the chemotactic responses of MSCs are generally considered to resemble those of immune cells. Consistent with this, inflammatory cytokines are strongly involved in modulating the mobilization of BM-MSCs in the BM niche, and the further trafficking and homing of those cells to tumor sites [33].

## 4. MDSCs and MSCs in T-Cell Immunosuppression Function and Mechanism

**Figure 1:**
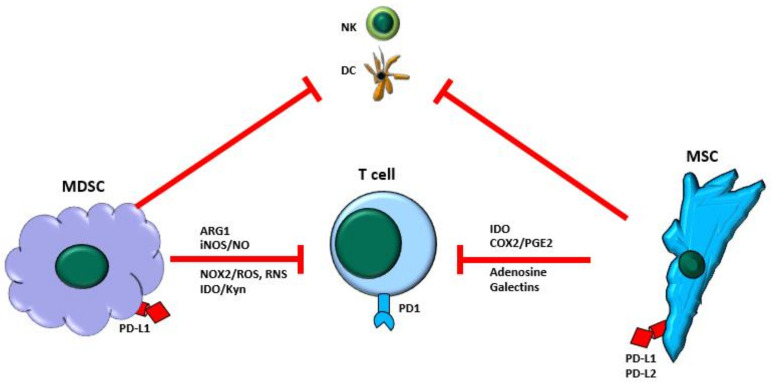
Overview of the immunosuppressive mechanisms of myeloid-derived suppressors cells (MDSCs) and mesenchymal stroma/stem cells (MSCs). Both MDSCs and MSCs exert a potent immune-suppressive function on components of the immune system, and they may express and release proteins and molecules that inhibit the activation and function of T lymphocytes (T-cells). In addition, MDSCs and MSCs may target other immune system cellular members such as dendritic cells (DC) and natural killer cells (NK). ARG1, arginase-1; iNOS, inducible nitric oxide synthase; NO, nitric oxide; COX2, cyclooxygenase-2; PGE2, prostaglandin-2; NOX2, NADPH oxidase-2; ROS, reactive oxygen species; RNS, reactive nitrogen species; IDO, indoleamine 2,3-dioxygenase; Kyn, kynurenine; PD1, programmed death-1; PD-L1, PD-L2, programmed death-ligand 1, -ligand 2.

MDSCs and MSCs have an immunoregulatory capacity of almost all immune system components that may affect cancer development during several stages [35,36]. For instance, MDSCs and MSCs intensely regulate the immune response via interactions with the innate system, such as natural killer cells and monocytes/macrophages, and adaptive immune systems, including DCs, B lymphocytes, and T lymphocytes. The dependent immunoregulation of MDSCs and MSCs occurs through cellular contact and the secretion of diverse factors (Figure 1) [18,37,38,39]. In inflammation, MDSCs and MSCs can prevent the inappropriate activation of T lymphocytes and generate a tolerogenic environment during wound repair or stop an immune response during healing, thus contributing to immune homeostasis maintenance. However, these properties can favor cancer development and allow transformed cells to escape from cancer immunosurveillance [37,40,41]. Next, we analyze the primary mechanism involved in the immunoregulation of T-cell activation, proliferation, and function by MDSCs and MSCs, which contributes significantly to the escape of transformed cells from anticancer immune responses.

### 4.1. MDSC and T-Cell Immunosuppression

MDSCs have been postulated to be highly immune-suppressive cells involved in endogenous and exogenous inflammatory insults [42]. Despite the capacity of MDSCs to regulate almost all immune cells, the inhibition of T-cells is critical, appears to be a sufficient functional criterion to define MDSCs, and is the gold standard for evaluating the function of MDSCs [14,17,43].

L-arginine catabolism may affect T-cell expansion. For instance, arginase-1 (ARG1) converts L-arginine (Arg) into ornithine and urea, whereas L-Arg is a precursor for nitric oxide (NO) production by inducible nitric oxide synthase (iNOS). L-Arg depletion provokes T-cell proliferation inhibition due to downregulation of CD3δ, cyclin D3, and cyclin-dependent kinase (CDK)4 expression [44,45,46]. Additionally, increased MDSC-associated iNOS/NO levels reduce T-cell proliferation by inhibiting interleukin (IL)-2 expression [47].

Interestingly, increased NADPH oxidase-2 (NOX2) expression in MDSCs is mainly regulated by the STAT3 transcription factor and contributes to elevated reactive oxygen species (ROS) levels, such as the superoxide anion (O2-) and hydrogen peroxide (H2O2) [48]. The O2- and free radical NO- reaction produces reactive nitrogen species (RNS), which drive T-cell apoptosis via T-cell receptor (TCR) tyrosine nitration [49]. In turn, T-cells’ TCRζ chain expression is inhibited by MDSC-derived H2O2 [50]. PMN-MDSCs primarily use ROS and RNS as the main mechanisms of immune suppression, which depend on tight cell–cell contact to affect T-cells due to these molecules’ short half-life [51]. By comparison, M-MDSCs suppress the function of T-cells by expressing increased amounts of NO, ARG1, and immunosuppressive cytokines, which does not require close contact between M-MDSCs and T-cells [52,53,54].

MDSC-induced T-cell immunosuppression within the TME also relies on IDO expression. IDO converts tryptophan (Trp) to active metabolites kynurenine (Kyn), kynurenic acid, and 3-hydroxykynurenine. Trp depletion induces cell cycle arrest and apoptosis in T-cells by inhibiting the mechanistic target of rapamycin complex 1 (mTORC1), whereas Kyn is toxic for lymphocytes by itself and contributes to the exhaustion of CD4+ T-cells [55,56,57]. In addition, MDSCs inhibit T-cell activation and induce apoptosis by a reduction in intratumoral cysteine levels. T-cells do not synthesize cysteine endogenously and depend on the uptake of DC-derived cysteine, whereas MDSCs sequester and reduce cysteine bioavailability through the cystine/glutamate transporter encoded by solute carrier family 7 member 11 (SLC7A11) [58,59]. 

Furthermore, MDSCs may induce the suppression of T-cell activation and apoptosis through cell–cell interaction mediated by immune checkpoint proteins, such as the programmed death-1 (PD-1) molecule with its cognate ligands PD-L1 and PD-L2 [60]. PD-L1 expressed on MDSCs binds T-cell-expressed PD-1 and inhibits T-cell functions [60,61]. 

In addition to impairment of T-cell activation, MDSCs also interfere with T-cell trafficking and restrict T-cells’ access to inflamed sites [62]. MDSCs provoke the cleavage of the ectodomain of the cell adhesion molecule L-selectin/CD62L by MMP17, which reduces cell surface L-selectin in T-cells and thereby limits their homing to peripheral lymph nodes and tumors [63]. Furthermore, MDSCs may decrease L-selectin expression in T-cells in an HMGB1-dependent fashion within the TME [64].

### 4.2. MSC and T-Cell Immunosuppression

As MSCs exhibit a low expression of HLA-II and costimulatory molecules, such as CD40, B7, CD80, and CD86, they do not stimulate alloreactive T lymphocyte responses *in vitro* and are considered to be hypoimmunogenic cells [65,66,67]. MSCs also regulate immune responses by influencing adaptive and innate immunity via soluble factors and cell-to-cell contact mechanisms [68,69]. MSCs mainly migrate to the sites of inflammation and display potent immunomodulatory and anti-inflammatory effects through cell–cell interactions with lymphocytes or by the production of soluble factors [67,68,69,70]. It is generally accepted that MSCs suppress T-cell proliferation, cytokine secretion, and cytotoxicity and regulate T helper (Th)1/Th2 effector cells’ balance [71]. Interestingly, MSCs also inhibit the lytic function of activated cytotoxic T-cells (CTLs) [72].

MSCs constitutively express and release indoleamine 2,3-dioxygenase (IDO), further enhanced by interferon (INF)-γ stimulation [73,74]. Consequently, Trp depletion inhibits allogeneic T-cell responses and IFN-γ production by Th1 cells while provoking IL-4 secretion by Th2 cells [70,75,76]. Trp depletion appears to be more critical for IDO-mediated T lymphocyte inhibition rather than kyn accumulation [77,78]. Remarkably, mammalian-derived MSCs can be classified into two main groups depending on IDO expression and iNOs for immunosuppression functions. Cytokine-licensed MSCs derived from several mammalian species demonstrate that monkeys, pigs, and humans employ IDO to suppress immune responses, whereas mice, rats, rabbits, and hamsters, belonging to the phylogenetic clade Glires, mainly employ iNOS [79,80].

Prostaglandin E2 (PGE2), synthesized by cyclooxygenase-2 (COX-2), regulates inflammation and the cancer evasion of immunity [81,82]. IL-6, TNF-α, and IFN-γ may increase PGE2 production by MSCs [83,84,85]. COX2 inhibition by indomethacin led to a substantial recovery of T-cell proliferation in bone marrow (BM), adipose tissue, and Wharton’s jelly-derived MSC co-culture approaches [86,87]. In addition, MSCs’ expression of COX2 and PGE2 production contributes to the maintenance of self-renewal and proliferation in an autocrine manner via the prostaglandin EP2 receptor [88].

MSCs constitutively express ectonucleoside triphosphate diphosphohydrolase-1 (CD39) and adenosine formation by ecto-5′-nucleotidase (CD73) to convert ATP into adenosine [89,90]. Adenosine functions as an immunosuppressant that binds to the adenosine A2a Receptor (ADORA2A), which triggers intracellular cAMP production to suppress T-cell proliferation and function, thus inducing T-cell anergy [91,92,93].

Galectins (Gal) are one of the main groups of carbohydrate recognition proteins that comprise the β-galactoside binding galectin family, and they are directly linked with immunity [94]. MSCs express Gal-1, Gal-3, and Gal-9, which induce apoptosis in activated T-cells [95,96,97]. 

In addition to the production of soluble factors, MSCs, via cell–cell interaction, may downregulate T-cell activation and responses; this occurs via induction of T-cell apoptosis through the inhibitory receptor interaction programmed death-1 (PD-1) protein with its cognate ligands PD-L1 and PD-L2 [71,98]. The co-culture of MSCs with allogeneic splenocytes stimulates, in conjunction with phytohemagglutinin, increased PD-L1 and PD-L2, whereas the use of specific blocking antibodies against PD-1, PD-L1, and PD-L2 allows splenocytes to proliferate, even in the presence of MSCs [99,100]. Furthermore, the combination of Interferon-γ and TNF-a increases placenta-derived MSC expression of PD-L2 and inhibits anti-CD3 antibody-stimulated T-cell proliferation, concomitantly with a switch of T-cell differentiation to the T regulatory (Tregs) cell phenotype. Moreover, the addition of anti-PD-L1 Mab protects T-cell proliferation of inhibition by placenta-derived MSCs [101,102].

## 5. MDSCs and MSCs in Myeloid Malignancies

**Figure 2:**
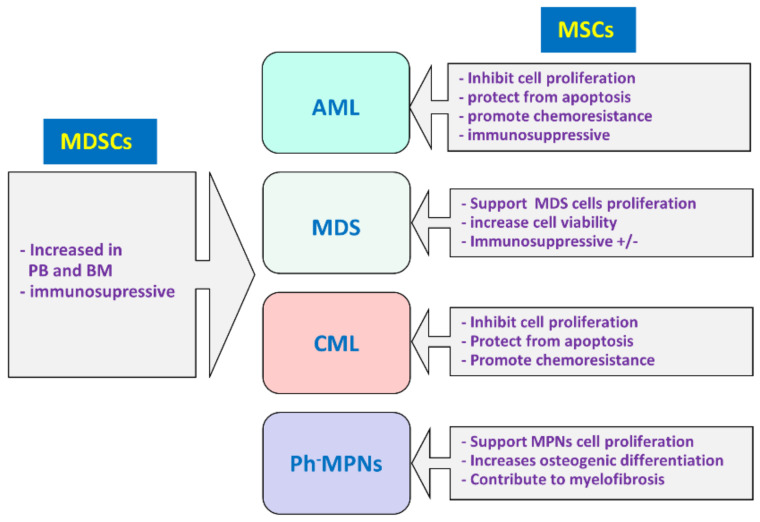
Main features of MDSCs and MSCs in myeloid malignancies. The number of MDSCs is elevated in peripheral blood (PB) and bone marrow (BM) of patients with myeloid malignancies, and they exhibit potent immunosuppressive functions. MSCs mainly support neoplastic myeloid cells and may promote chemoresistance; depending on the myeloid malignancy subtype, they may exert specific functions, as indicated in the figure. AML, acute myeloid leukemia; MDS, myelodysplastic syndrome; CML, chronic myeloid leukemia; Ph-MPN, Philadelphia chromosome-negative myeloproliferative neoplasms.

Myeloid malignancies arise from immature myeloid clones due to genetic mutations, which alter proliferation, maturation, and differentiation in a process called clonal hematopoiesis [103]. Malignant transformation can appear at any stage of blood cell development, including HSCs, progenitors, and mature blood cells [104]. According to the 2017 revision of the World Health Organization (WHO) classification of myeloid malignancies, myeloid malignancies include BCR-ABL1/Philadelphia (Ph), positive and negative MPN, MDS, MDS/MPN with mixed features, myeloid and lymphoid neoplasms associated with eosinophilia, gene rearrangements, and AML [105,106]. Next, we analyze the main features of MDSCs and MSCs in myeloid malignancies. Figure 2 indicates the main features of MDSCs and MSCs in myeloid malignancies.

### 5.1. Acute Myeloid Leukemia

AML is the most common myeloid malignancy and is highly aggressive. AML is a heterogeneous clonal disorder characterized by the expansion of immature myeloid progenitors (blasts) in the BM and peripheral blood [107]. AML development is the outcome of hematopoietic progenitor transformation leading to the accumulation of rapidly proliferating, abnormal myeloid cells incapable of terminal differentiation [108]. In this type of leukemia, the highly controlled cell proliferation process and differentiation are disturbed by mutations that alter the function of growth factors, their receptors, and intracellular signal transduction [109]. 

AML develops due to oncogenic activation in myeloid progenitors in the bone marrow and is characterized by blood tissue destruction, which causes acute pancytopenia, severe bleeding, and infection [110]. The most common mutation in AML, and a poor prognosis marker, is a mutation in the FLT3 gene (FMS-like tyrosine kinase 3). A gain-of-function mutation in the FLT3 gene leads to activation of signal pathways, such as MAPK, STAT, and AKT, which are essential for dysregulated cell proliferation and apoptosis resistance. Moreover, immune evasion is also a critical characteristic of AML cells [111]. Over-expression of PD-L1, T-cell anergy, Tregs accumulation, and MDSCs are vital factors in the AML antitumor immune response [112]. Moreover, systematic analyses of AML progression mechanisms are key to the development of new therapeutic approaches in AML.

#### 5.1.1. MDSCs in Acute Myeloid Leukemia

Patients with AML have an increased level of CD14^−^HLA^−^DR^−^CD33^+^ CD11b^+^ MDSCs in both PB and BM. A significant correlation exists between PB-associated MDSCs and conventional prognostic factors at diagnosis, whereas BM-associated MDSCs may impact the disease prognosis and the AML patient’s clinical course [113]. In addition, in de novo diagnosed AML patients, the frequency of MDSCs correlates with the subtype of AML, chromosomal and gene modifications, and D-dimer plasma levels. Furthermore, after therapy and complete remission, patients exhibit a decrease in the number of MDSCs compared to partial or nonresponsive patients, whereas the frequency of MDSCs correlates with minimal residual disease levels [113]. Moreover, a high number of M-MDSCs in the PB of AML patients is associated with a poor prognosis, with potential use as a prognostic indicator of the disease [114]. Furthermore, the high levels of PB-associated eMDSCs, such as CD33^+^CD11b^+^ HLA-DR^−/low^CD14^−^CD15^−^ cells, in AML patients indicate a potential use as a diagnostic index [115]. 

Furthermore, a high frequency of BM-associated CD11b^+^ CD33^+^ HLA^−^DR^−^ MDSC-like blasts is associated with a significantly shorter overall survival and poor prognosis, whereas a low number of MDSC-like blasts may indicate leukemia-free survival of AML patients. Moreover, MDSC-like blasts may drive AML to escape from immune control by suppressing CD8^+^ T-cell proliferation by ARG1 and iNOS expression [116]. 

Similarly, M-MDSCs in newly diagnosed AML patients exhibit immunosuppressive functions due to the expression of IDO. Interestingly, AML-derived extracellular vesicles (EVs) induce monocytes to acquire an M-MDSC (CD14^+^HLA-DR^low^) phenotype. Specifically, palmitoylated proteins on the AML-EV surface activate Toll-like receptor 2 in monocytes and trigger an Akt/mTOR-dependent induction of MDSCs [117]. AML-derived EVs also induce MDSCs from PB mononuclear cells (PBMCs). It appears that mucin 1 (MUC1) oncoprotein promotes EV-associated c-myc expression, which, when taken up by PBMCs, induces cyclin D2 and E1 and selectively enhances MDSC proliferation [118]. This suggests that AML-transformed cells may contribute to the expansion of MDSCs, sustaining the immunosuppressive characteristics observed in AML patients.

#### 5.1.2. MSCs in Acute Myeloid Leukemia

MSCs appear to have an antiproliferative function on AML cells. For instance, the human bone marrow stromal cell line (HFCL) induces a G_1_ cell cycle arrest of AML cell lines, such as U937, HL-60, and multidrug-resistant HL-60/VCR cell lines. Moreover, HFCL co-culture protects AML cells from apoptosis induction by topoisomerase I (topo I) inhibitor topotecan (TPT) and, as observed by Annexin-V assay, activates a Caspase-3 decrease and increases Bcl-2 expression [119]. Similarly, umbilical cord-derived MSCs inhibit cell proliferation and cell arrest at G_0_/G_1_ by expressing and releasing IL-6 and IL-8 [120]. As MSCs induce AML cell quiescence and protect cells from apoptosis, an increment in chemoresistance is observed. AML-increased chemoresistance appears to be associated with the increment of c-myc and B-cell lymphoma (Bcl)-2 expression and Notch signaling [121,122,123]. Furthermore, MSCs exert an antiapoptotic and growth-enhancing effect on primary human AML cells by mTOR signaling pathway activation [124].

Direct MSC contacts are mainly required to acquire AML cells’ drug resistance, as demonstrated by Garrido et al. by co-cultivating derived leukemic cells from AML patients with HS-5 human BM stromal cell monolayers [125]. AML cells in contact with HS-5 monolayers exhibit resistance to apoptosis induction by cytosine arabinoside or daunomycin treatment, whereas noncontact conditions inhibited drug-induced apoptosis of AML cells. Moreover, the reciprocal VCAM-1/VLA-4-dependent NF-κB activation in MSCs and AML cells mediates the stromal cell-mediated drug resistance in leukemia cells *in vitro* and *in vivo* [126].

AML-derived MSCs (AML-MSCs) exhibit some differences compared to MSCs from healthy donors; although AML-MSCs express CD90, CD73, and CD44 levels that are similar to those of a healthy counterpart, a decrease in chemoattractant protein-1 levels is observed [127]. Moreover, AML-MSCs possess an enhanced capacity to support hematopoiesis due to an abnormal expression of cell surface molecules, such as CD44, CD49e, CD271, and CXCL12 [128,129]. In addition, about 25% of AML-MSCs display genetic aberrations, the most frequent of which are chromosomic translocations, which are different from those in hematopoietic cells [130]. Moreover, AML-MSCs display several gene mutations, such as those found in plectin and chromatin remodeling genes, and hypermethylation of pituitary homeobox (PITX)2 and Homeobox (HOX)B6 genes and hypomethylation of HOXA3 and HOXA5 genes have also been described [131,132].

In addition to AML-MSCs’ genome modifications, cell functional changes have been reported. Controversial information can be found in the literature indicating that AML-MSCs exhibit reduced clonogenic potential (CFU-F) at diagnosis, reduced proliferation capacity, and susceptibility to enter the senescence stage. In contrast, CFU-F potential is restored in AML-MSCs from patients in complete remission [132,133]. Conversely, AML-MSCs derived from patients from good-, intermediate-, and poor-risk groups exhibit an increase in clonogenic potential [134]. Furthermore, these AML-MSCs display an enhanced immunosuppressive capacity that is probably associated with increased anti-inflammatory IL-10 production, which correlates directly with patients’ overall survival, whereas AML-MSCs have reduced proinflammatory cytokine expression [134]. Interestingly, MDS-derived MSCs (MDS-MSCs) may transfer functional mitochondria to AML cells *in vivo*, which increase AML cells’ energy production and cell survival under chemotherapy conditions [135,136].

### 5.2. Myelodysplastic Syndrome

MDS is another clonal HSC disorder characterized by dysplasia of myelopoiesis and consequential pancytopenia. Classification of MDS is based on the quantity of dysplastic myeloid cell linages (single-lineage and multilineage MDS), presence of ring sideroblasts (RS) (RS-MDS), chromosomal aberrations (MDS with del5q), or blasts in the bone marrow and peripheral blood (MDS with excess blasts) [137,138,139]. Due to enhanced apoptosis, increased phagocytosis, and reduced cell differentiation, cytopenia may predispose patients to potentially life-threatening complications, such as bleeding or infections, which are the most common causes of death associated with MDS [140,141,142]. MDS is the most common myeloid hematologic malignancy and mainly affects the elderly population. About 30–45% of patients may progress to acute myeloid leukemia [143,144]. 

Patients with MDS, particularly those with excessive blast expression, carry a high risk of AML transformation. Genetic abnormalities such as del5q, del7q, t(7;17), mutated NRAS, and mutated RUNX1 genes can be detected at disease diagnosis but are more common at disease progression. Recent studies demonstrated their importance for diagnosis, classification, drug response, and prognosis of the disorder [145,146]. An essential feature in MDS disease pathogenesis is inflammation and immune dysregulation [147]. MDS progenitors are prone to apoptosis due to over-expression of death receptors (Fas and TRAIL-R) and impaired caspase activity [148]. Over-expression of suppressive cytokines tumor necrosis factor (TNF)-α, transforming growth factor (TGF)-β, and IFN-γ, over-expression of TLR, and hyperactivation of cytotoxic CD8+ T lymphocytes have been reported in MPN [149]. In addition to some shared molecular features, including chronic inflammation and molecular mutations, MDS and MPN also have some common clinical characteristics; thus, the new modality MDS/MPN with mixed features in the WHO classification was inevitable [106].

#### 5.2.1. MDSCs in Myelodysplastic Syndrome

MDSCs are significantly increased in the BM and PB of high-risk MDS compared to lower-risk MDS patients [150,151,152], and an elevated MDSC BM frequency may promote ineffective hematopoiesis concomitantly with increased immunosuppression in T-cells [153]. Moreover, the increased CD33 expression contributes to the dysfunctional myeloid cell development, and CD33 binding to S100A9 promotes MDSC expansion and induces anti-inflammatory IL-10 and TGF-β cytokine secretion. Furthermore, CD33 knockdown reduces IL-10 and TGF-β secretion and ARG1 activity, therefore downregulating MDSC immunosuppressive activity; moreover, the induction of MDSCs’ maturation, by either all-trans-retinoic acid treatment or active immunoreceptor tyrosine-based activation motif-bearing (ITAM-bearing) adapter protein (DAP12) interruption, rescues the hematologic phenotype of MDS [153,154]. In addition, high-risk MDS-derived MDSCs exhibit higher activated activator of transcription (STAT)3 and C-C chemokine receptor type (CCR)2 expression, whereas STAT3 pathway targeting decreases ARG1 expression in MDSCs and partially revokes reduced expression levels of effector molecules in CD8+ T lymphocytes [155]. Thus, MDSCs may contribute to the BM microenvironment switch to an immunosuppressive environment as the MDS disease progresses [156].

#### 5.2.2. MSCs in Myelodysplastic Syndrome

Increased MSC cell density in the BM of higher-grade MDS compared to lower-grade MDS and benign hematologic disorders independently correlate with significantly shorter overall survival [157]. MDS-MSCs exhibit a reduced *in vitro* proliferation capacity compared to healthy counterparts, which may indicate the intrinsic growing defect of MDS-MSCs, although these cells show similar immunophenotype patterns and differentiation capacity to those displaying normal MSCs. MDS-MSCs support leukemic cell proliferation and viability similar to those of healthy MSCs [158]. MDS-MSCs possess increased clonal hematopoiesis-supportive capacities due to a decreased expression of cell surface molecules, including CD44 and CD49e (α5-integrin), and lower or absent expression levels of costimulatory molecules (such as CD40, CD80, and CD86) [128,159,160,161]. In particular, MDS-MSCs are epigenetically and functionally altered, resulting in deficient support of normal hematopoiesis [132].

MDS-MSCs *in vitro* display modification of expression cytokines, such as decreased expression of stem cell factor (SCF), granulocyte cell stimulating factor (G-CSF), and granulocyte-macrophage colony-stimulating factor (GM-CSF), and increased expression of IL-6 [162]; adhesion molecules, such as CD44 adhesion molecules and CD49e [163]; molecules involved in the interaction with the HSCs, such as osteopontin (OPN), Jagged1, Kit-L, and angiopoietin (ANG)1 [164]; and the CXCL12 chemokine associated with the survival/antiapoptosis of MDS cells and disease progression in MDS [165]. Moreover, the clonogenic potential and proliferation of MDS-MSC CFU-Fs are reduced at diagnosis [166,167,168], and they exhibit accelerated senescence earlier than in healthy MSCs [169]. Moreover, MDS-MSCs inhibit *in vitro* erythroid hematopoiesis and promote the myeloid cell lineage of HSCs *in vitro* [164].

MDS cells may modify MSCs’ biological behavior, as demonstrated by the capacity of conditioned medium of human MDS cells to educate healthy MSCs to acquire MDS-MSC molecular features. For instance, methylation signatures, upregulation of cytokine/inflammation-related genes, and downregulation of cell cycle-promoting genes reduce the supportive capacity of healthy HSCs [132,170]. Some studies have indicated that patients’ MDS-MSCs appear to have an impaired immunoregulatory function [171,172], whereas others showed that the immunosuppressive function did not differ significantly between patients and healthy MSCs [167]. Furthermore, MDS-derived MSCs exert an immunosuppressive effect due to increased prostaglandin production, which may reduce T-cell immunity against leukemic cells [173].

### 5.3. Myeloproliferative Neoplasms

MPNs are characterized by the clonal proliferation of one or more hematopoietic cell lineages, predominantly in the bone marrow, and demonstrate terminal myeloid cell expansion into the peripheral blood [174]. MPNs mainly include CML, polycythemia vera (PV), essential thrombocythemia (ET), primary myelofibrosis (PMF), and unclassifiable MPNs [175].

#### 5.3.1. Chronic Myeloid Leukemia

CML is a malignant myeloproliferative disorder characterized by clonal hematopoietic stem cell proliferation [176]. CML is the BCR-ABL1 oncoprotein-positive MPN characterized by the Philadelphia (Ph) chromosome’s presence. The Ph chromosome, a unique biomarker of CML, is a product of reciprocal translocation of the BCR gene on chromosome 22q11.2 and the ABL1 gene located on chromosome 9q34, and about 90–98% of CML patients harbor this mutation [105,177,178]. Furthermore, CML incidence is about 0.7–1.0/100,000 individuals per year; this rate has stabilized in recent years, and the diagnosis is more frequent in the population around 60–70 years old [179].

The BCR-ABL1 oncogene leads to the constitutive activation of tyrosine kinase, an essential factor in leukemogenesis and target molecule for CML treatment with tyrosine kinase inhibitor (TKI). As a consequence of cytokine-independent tyrosine kinase hyperactivity, several signal pathways, such as rat sarcoma (RAS) protein, mitogen-activated protein kinases (MAPK), and PI3K/AKT, are activated, leading to an improved proliferation of leukemic progenitor cells and apoptosis evasion [180,181]. Although BCR-ABL leads to unrestricted cell proliferation, dysfunctional cell differentiation, and apoptosis resistance, the advances in the development of TKIs, as part of the armamentarium of innovative treatments, increase the therapeutic success rates and substantially increase patient survival and disease prevalence [177,182,183]. Nonetheless, the sustained CML stem cell proliferation may favor generating new mutations that provoke resistance to the current treatments and negatively impact disease prognosis [184]. Disease progression and resistance to TKI are associated with mutations of tumor protein (TP)53, MYC, and KRAS genes, MSC-mediated protection of leukemic cells, accumulation of MDSC, disrupted function of NK cells, and cytotoxic T lymphocytes [185,186,187]. Overall, these findings highlight the importance of immunomodulation and “oncoinflammation” in the development of new treatment strategies.

MDSCs in chronic myeloid leukemia.

An increase in the number of MDSCs has been found in the PB and BM of CML patients [188,189]. An elevated frequency of PMN-MDSCs has been observed in CML and may contribute to CML cells’ escape from immune surveillance [188,190]. Imatinib treatment reduces and recovers the basal levels of PMN-MDSCs in CML patients, whereas the frequency of M-MDSCs may be used as a prognostic factor for dasatinib therapy in CML patients [191,192]. Furthermore, CML patients responsive to TKI therapies, such as imatinib, nilotinib, and dasatinib, show a decrease in the number of M-MDSCs concomitant with a reduced immunosuppressive function in T-cell and NK cells, in conjunction with ABL-BCR1 transcript reduction [187]. In addition, an increased ARG1- and PD-L1-expressing PMN-MDSC frequency is also observed in Sokal high-risk CML patients and T-cells with an upregulated PD-1 expression [190]. Moreover, the number of M-MDSCs may correlate with CML patients’ remission status. In addition, they promote the *in vitro* proliferation of the K562 cell line and CD34+ cells obtained from newly diagnosed CML patients [189], which suggests that MDSCs, in addition to their immunosurveillance protection, may increase the number of CML cells and exacerbate the development of CML disease [193].

MSCs in chronic myeloid leukemia. 

MSCs play essential roles in CML cell growth, apoptosis, and resistance to chemotherapy. For instance, BM-MSCs protect CML cells from imatinib-induced cell death, mediated by MSC-secreted IL-7 and activation of the JAK1-STAT5 pathway CML cells. Moreover, IL-7 levels are increased in the BM of CML patients in the blast crisis phase [194,195]. Although imatinib enhances CXCR4 expression in CML cells, and MSCs protect these CML cells from imatinib-induced apoptosis, MSCs further enhance the capacity of imatinib to induce CML cell cycle arrest in the G_0_/G_1_ phase. Thus, imatinib may enhance CML cells to migrate to BM by increasing CXCR4 expression and block cell proliferation; BM stromal cells may protect and promote quiescent CML survival cells with potential implications for disease re-incidence [196,197].

Similarly, MSC contact inhibits apoptosis and promotes a G_0_/G_1_ quiescent state of the Ph-positive human cell line (BV173) and K562 cells coupled with cyclin-D2 downregulation. Conversely, MSCs promote tumor growth of CML cells in NOD-SCID mice with the expected apoptosis inhibition. These results suggest to the authors that MSCs may contribute to a niche creation of cancer stem cells that preserve CML stem cells’ self-renewal and sustain malignant processes [198]. MSCs may, *in vitro*, inhibit CML mononuclear cell proliferation due to the enhanced production of interferon (IFN)-α in co-cultured conditions; IFN-α was the standard frontline treatment for chronic myeloid leukemia prior to TKIs [199,200]. In addition, BM-MSCs obtained from the blastic phase (Bp) of CML patients *in vitro* protect primary CML Bp cells from apoptosis induced by Adriamycin via reduction in caspase-3 and Bax expression, an increase in Bcl-2 levels, and activation of the Wnt pathway [201].

#### 5.3.2. Philadelphia Chromosome-Negative Myeloproliferative Neoplasms 

Ph- MPNs are a group of hemopoietic stem cell disorders characterized by clonal proliferation of myeloid-lineage cells in the bone marrow and chronic inflammation [202]. This group of disorders is encompassed by polycythemia vera (PV), which typically display a high number of red cells, usually in conjunction with thrombocytosis and essential thrombocythemia (ET), characterized by megakaryocyte expansion and increased platelet count. The main features of primary myelofibrosis (PMF) are peripheral leuko-erythroblastosis, massive splenomegaly, and BM fibrosis [202,203,204]. ET or PV may potentially evolve into either end-stage myelofibrosis with BM failure or the development of secondary acute leukemia [205]. PV and ET have an incidence of 0.5 to 4.0 and 1.1 to 2.0 cases per 100,000 person-years, respectively, with similar survival predictions. PMF, in turn, is less frequent, presenting an incidence of 0.3 to 2.0 per 100,000 person-years, but is associated with the shortest survival of the MPNs [206]. 

The mutational landscape of MPNs is formally integrated into the WHO diagnostic criteria for PV (98% JAK2 mutational frequency), ET (50–60% JAK2, 22% calreticulin (CALR), and 3% thrombopoietin receptor (myeloproliferative leukemia virus, MPL), and PMF (50–60% JAK2, 25% CALR, and 7% MPL). Moreover, about 10–15% of patients with PMF or ET lack these three driver mutations and are referred to as triple-negative MPN [202,207]. There are more than 20 other rare mutations responsible for disease progression and transformation, comprising ASXL1 (additional sex combs-like 1), IDH1/2 (isocitrate dehydrogenase 1/2), TP53, and TET2 (TET oncogene family member 2) [208].

Clonal myeloproliferation in MPN is the consequence of the chronic inflammation activated by infection, hypoxia, or injury. Cytokine production is initiated by activation of signal pathways such as nuclear factor (NF)-κB, STAT1, STAT3, or hypoxia inducible factor (HIF)-1α, or is the result of MPN-associated mutations [209]. Dysregulation of the immune system is another critical feature of MPN. Accumulation of CD4+CD25+ forkhead box (FOX)P3+ Tregs, monocytes, macrophages, and MDSCs, and an impaired balance of CD4+/CD8+ have been hypothesized as relevant factors for disease progression and therapeutic resistance [210]. Other atypical MPNs comprise chronic neutrophilic leukemia, chronic eosinophilic leukemia, mastocytosis, and rare clonal disorders characterized by persistent neutrophilia, eosinophilia, or accumulation of mast cells [105].

MDSCs in Ph- myeloproliferative neoplasms.

Ph- MPNs appear to dysregulate the function of immune cells, which includes an increased number of monocyte/macrophages, dysfunctional NK cells, and expansion of MDSCs [210]. Peripheral blood MDSCs are significantly elevated in MPNs without differences in their frequency between the different MPN types, and no correlations with JAK2 allele burden have been found. Consistently, MPN-derived MDSCs exhibit an immunosuppressive function by inhibiting the proliferation of autologous CD3+ T-cells alongside elevated ARG1 expression. Moreover, the frequency of MDSCs is likely increased in the BM of MPN patients, although their function and activity remain to be elucidated [211].

MSCs in Ph- myeloproliferative neoplasms.

MSCs appear to support transformed myeloid proliferation and favor the creation of a BM fibrotic environment. For example, in a primary myelofibrosis in a thrombopoietin (Tpo)-over-expressing mouse model, MSCs expressing the leptin receptor are the primary source of myofibroblasts in BM or PMF [212]. In addition, although MPN (JAK2^V617F^)-derived MSCs do not exhibit differences in morphology, proliferation, and differentiation capacity compared to healthy counterparts, they better support the CFU-GM clonogenicity of MPN-hematopoietic stem/progenitor cells. Consistently, MPN-MSCs derived from PV and ET patients exhibit a modified expression of genes associated with hematopoiesis maintenance, such as secreted phosphoprotein (SPP)1 and NF-κB over-expression, and angiopoietin (ANGPT)1 and thrombopoietin (THPO) downregulation. Thus, MPN (JAK2^V617F^)-derived MSCs may favor the expansion and maintenance of MPN cells [213]. Schneider et al. also found that ET- and PV-derived BM-MSCs are similar to healthy MSCs in terms of surface marker pattern, CFU-F clonogenicity, morphology, and differentiation capacity. Intriguingly, ET-MSCs secreted significantly lower G-CSF and IL-7, which indicates a potential impairment in the hematopoiesis-supporting capacity, whereas normal myeloid CFU is reduced when cultivated with supernatants from PV cells [214]. In addition, PMF-BM-MSCs display no differences in proliferation or capacity to support normal hematopoiesis compared to healthy BM-MSCs. Nonetheless, PMF-, PV-, and ET-BM-MSCs appear to be preferentially reprogramed to be committed to osteogenic differentiation compared to normal BM-MSCs, and consequently to upregulation of runt-related transcription factor (RUNX)2, distal-less homeobox (DLX)5, OPN, and integrin binding sialoprotein (IBSP) expression [215]. Furthermore, MPN-BM-MSCs promote an abnormal generation of osteoblast-like inflammatory “myelofibrotic” cells, as a result of a dysregulated inflammatory milieu due to elevated TGF-β1, Notch, IL-6, IL-1β, and TNF-α production, which may be a consequence of direct contact between MPN-HSCs and BM-MSCs [216]. Moreover, excessive expansion of inflammatory “myelofibrotic cells” favors the progressive BM fibrosis generated in the advanced stages of MPN; in addition, over-generation of osteoblasts contributes to perpetuating clonal-MPN cell proliferation [217,218]. 

Interestingly, a consistent reduction in sympathetic nerve fibers, supporting Schwann cells and nestin^+^ MSCs, is observed in the bone marrow of MPN patients and mice expressing the JAK2(V617F) mutation in HSCs. Consequently, the abrogation of bone marrow nestin+ MSCs innervated by sympathetic nerve fibers is essential for MPN pathogenesis. Furthermore, the restoration of sympathetic regulation of nestin+ MSCs by β3-adrenergic agonists prevents mutant myeloid cell expansion [219]. In addition, elements of the PV-derived BM-MSC secretome, including IL-6, G-SCF, and CXCL-10/IP-10 production, contribute to therapy resistance to JAK2 inhibition and reduce JAK2(V617F) myeloid cell apoptosis in co-culture conditions with MSCs *in vitro* [220].

## 6. MDSC and MSC Interplay

Recently, MSCs have been proposed to be a new actor in the generation and accumulation of MDSCs, which highlights the complex tumor stroma scenario. BM-MSCs have been shown to induce human CD14−CD11b+CD33+ MDSC expansion and function *in vitro* and via paracrine effects through hepatocyte growth factor (HGF). BM-MSC-secreted HGF interacts with its cell surface receptor c-met in peripheral blood mononuclear cells, triggering the activation of STAT3 in conjunction with increased levels of iNOS and ARG1, which mediate the immunosuppressive activity of generated MDSCs in T-cell proliferation. Furthermore, as MDSCs inhibit T-cell activation, they shift their fate to CD4+CD25highCD127low Tregs, which further enhances immunosuppression [221]. Growth-regulated oncogene (GRO) chemokines, such as GRO-γ, secreted by MSCs, may expand MDSCs. GRO-γ present in MSC medium shows the capacity to induce a phenotype switch of monocyte-derived dendritic toward an MDSC-like phenotype. GRO-γ-induced MDSCs express a tolerogenic phenotype characterized by increased secretion of IL-10 and IL-4, and ARG1 and iNOS expression, whereas production of IL-12 and IFN-γ is reduced [222].

MSCs appear to play a role in the induction of MDSCs in multiple myeloma (MM). Although MSCs derived from healthy donors, monoclonal gammopathy of uncertain significance (MGUS), and MM generate a similar number of MDSCs *in vitro*, only dysfunctional MM-derived MSCs generate PMN-MDSCs with immunosuppressive capacity. This result correlates with the increased frequency of PMN-MDSCs observed in MM patients. MM-derived MSCs secrete TGF-β, IL-10, and IL-6 levels that can mediate PMN-MDSC expansion. Furthermore, these MM-MSC-generated PMN-MDSCs exhibit a digestive process of bone, which may be related to enhanced bone resorption in MM patients [223,224].

As mentioned above, the frequency of PMN-MDSCs is elevated in CML patients, which may be explained, in part, by the capacity of CML-derived MSCs to promote *in vitro* the expansion of PMN-MSCs with immunosuppressive activities from peripheral blood mononucleated cells. The increased upregulation of immunomodulatory factors, such as TGF-β, IL6, and IL-10, in CML-derived MSCs, correlates with their capacity to promote PMN-MDSC generation and cell reprogramming, which occurs in conjunction with their increased expression of immunosuppressor enzymes, such as ARG1 and COX-2. Thus, CML-MSCs may directly orchestrate immune escape by driving MDSC activation in the tumor microenvironment in CML patients [188]. Overall, targeting dysfunctional MSCs may indirectly reduce MDSC frequency in cancer patients and restore the T-cell-mediated immunosurveillance, thus improving patients’ long-term outcomes. Nevertheless, the number of investigations into the interplay of MSCs and MDSCs in hematologic malignancies is scarce, and new studies are necessary to derive a general picture of the molecular and biological interaction of these non-transformed cells in malignancies. Finally, MDS-MSCs educate monocytes to display M-MDSC features. These MDSCs exert immunosuppression of T-cells and inhibition of NK cells’ function capacity via membrane-bound TGF-β and express elevated ROS levels through upregulation of ectodermal-neural cortex 1 (ENC1) expression, an inhibitor of the transcription factor Nrf2. Thus, MDS-derived MSCs, in part, may promote the expansion of MDSCs that is observed in the BM of MDS patients [152,225].

At present, only separate information of MDSCs’ and MSCs’ roles in Ph- MPNs can be found in the existing literature data. Therefore, it is necessary to conduct new investigations addressing the mutual collaboration of MSCs and MDSCs in these groups of hematopoietic stem cell disorders, which may improve the understanding of microenvironment roles in the etiology of Ph- MPN malignancies.

## 7. Concluding Remarks

There is convincing evidence regarding the contribution of inflammation in the pathogenesis of myeloid malignancies. Inflammation promotes the initiation and progression of myeloid malignancies and induces immunosuppression by inhibiting the adaptive and innate immune systems [42,226]. The significant advances made in recent decades have revealed the crucial roles of the BM microenvironment in myeloid malignancies’ pathogenesis. However, the underlying biological and molecular mechanisms have recently started to be unveiled and show a high level of complexity in all myeloid malignancies and disease stages. The primary function of MDSCs may prevent excessive inflammatory responses, but in a dysregulated inflammatory milieu, created by malignant cells, they may promote a permissive BM microenvironment that protects transformed cells, and they can survive, proliferate, and escape from immune surveillance and antitumor therapies [12,227]. In addition, inflammation may alter the BM niche to promote myeloid malignancies’ progression and confer chemotherapy resistance [228]. BM-MSCs, as essential BM-niche non-hematopoietic cells involved in structure formation and organization of the microenvironment, are also influenced by neoplasm cells and secreted inflammatory factors, which may engage an “inflammatory loop” that affects healthy HSCs and modifies normal hematopoiesis [229,230].

Although myeloid malignancies encompass several heterogeneous neoplasms, some common features can be defined after the analysis of MSCs’ and MDSCs’ roles in these groups of myeloid disorders (Figure 2): in general, in myeloid neoplasm, the frequency of MDSCs is elevated in PB and BM, exhibits potent immunosuppressive functions, and may indicate poor prognosis. In addition, MSCs, presumably in the BM niche, regulate myeloid neoplastic cell proliferation and increase their survival by affecting apoptosis induction, which results in chemotherapy resistance because MSCs provide a protective microenvironment for transformed cells. Moreover, MSCs also exert immunosuppression, which further contributes to an immune-tolerant BM microenvironment. The role of MDSCs and MSCs in myeloid neoplasm’s pathogenesis makes them attractive targets for generating new therapeutic strategies that may improve the current therapies. T-cell-engaging bispecific antibodies (bsAbs) are a promising tool for cancer treatment. For instance, in preclinical studies in AML patients, with the aim of achieving combinatory eradication of MDSCs and redirection of T-cells against AML-blasts by using a CD33/CD3-bispecific BiTE^®^ antibody construct (AMG 330), a reduction in IDO^+^ CD33^+^ MDSCs has been observed with boosted AML-blast lysis, thus suggesting that AMG 330 exhibits anti-leukemic efficacy by improving T-cell-mediated cytotoxicity and simultaneous MDSC depletion [231]. The low immunogenicity and limited recognition by HLA-incompatible hosts make MSCs an excellent choice for cellular therapy for delivering anticancer agents. Moreover, MSCs tend to accumulate close to malignant lesions. MSCs engineered to express CD33-CD3 bispecific antibodies prevent the establishment of AML in a NOD/SCID IL2Rγ−/− (NSG) mouse model and retargeting of autologous T-cells towards blasts obtained from AML patients [232]. Although MDSCs were not included in this study, as mentioned above, MSC-expressing CD33-CD3 bispecific antibodies may also reduce the frequency of MDSCs in AML patients.

Interestingly, TGF-β1 induces healthy MSCs to develop a dysfunctional phenotype and adopt a phenotype similar to that observed in myeloid neoplasm patient-derived stromal cells. For example, in AML/MDS-MSCs, treatment with SD-208, an inhibitor of autocrine and paracrine TGF-β signaling [233], abrogates the suppressive effects of TGF-β1 on stromal cell functionality and restores the osteogenic differentiation capacity of patient-derived stromal cells [234]. In addition, TGF-β1 induces MDSC expansion and immunosuppressive function. Moreover, MDSCs secrete copious amounts of TGF-β, and this cytokine level is elevated in myeloid neoplasm [235,236]; it is plausible that TGF-β1 inhibition may also considerably reduce the number of MDSCs and simultaneously restore the MSC function in the BM niche. Moreover, due to its solid immunosuppressive function, the TGF-β1 block may also promote T-cell function [237].

In addition, immune dysregulation in myeloid neoplasm is an attractive approach for immunotherapies, and an increasing number of studies support the use of immune checkpoint blockers, vaccines, and adoptive T-cell therapies to boost specific T-cells’ anticancer functions in myeloid malignancies. Immune checkpoints are a protective immune mechanism that dampens T-cell antigen responses of activated T-cells. Indeed, immune checkpoint inhibitors (ICIs) can enhance killer cells’ cytotoxicity against myeloid leukemic blasts [238]. Furthermore, the immune checkpoint PD-1 is induced in activated T-cells, and the binding with PD-L1-expressing cells provokes T-cell anergy [239]. The use of anti-PD-1 and anti-PD-L1 blocking antibodies has shown extraordinary results in solid tumors and hematological disorders [240,241]. PD-1-blocking antibodies have been tested in MDS and AML, especially in combination with the hypomethylating agents, such as 5-aza-2′deoxycitidine, which have shown promising results in relapsed/refractory AML [242].

Moreover, the oncogene-driven JAK2^V617F^ mutant cells in MPNs promote PD-L1 expression and immune escape [243]. Several clinical trials have addressed the safety and efficacy of ICIs in the setting of MPNs [reviewed in 204]. Although the MDSC and MSC interaction in MPNs has not yet been addressed, both types of cells are also a target for ICIs, since they express PD-L1/PD-L2 that results in downregulation of T-cell activation and induction of T-cell apoptosis [60,61,71,98]; thus, ICI immunotherapy also may restrain the immunosuppressive activities of MDSCs and MSCs and enhance the cytotoxic T-cell killing function in malignant myeloid cells.

Another attractive immunotherapy for myeloid malignancy treatment involves using chimeric antigen receptor T (CART) cells [244,245]. CART cell immunotherapy requires a genetic patient-derived T-cell modification to express a specific CAR, and a subsequent ex vivo cell expansion and reinfusion back into the same patient to eliminate neoplastic cells. CARs are mainly bio-synthetic receptors comprising an extracellular domain that expresses a single-chain variable fragment (scFv) derived from an antitumor antigen–antibody, a transmembrane domain, and an intracellular T-cell activation and co-stimulation signaling domain primarily composed of CD3ζ, CD28, and/or 4-1BB [246]. CART strategies are conducted in AML targeting CD123 (IL-3Rα) expressed in a subset of myeloid progenitors and widely found in hematologic malignancies [247]. CD123-CART cells show antitumor activity in CD123^+^ AML cell lines and primary patient AML cells *in vitro* and *in vivo*, and CD123-CART cells have limited toxicity in normal BM HSPCs, indicating a safety profile. Moreover, CD123 CART therapy showed remissions of AML and acceptable feasibility and safety in the first-in-human clinical trial [248,249,250]. In addition, CART cells target IL1 receptor-associated protein (IL1RAP) in quiescent CML stem cells. IL1RAP is a potential biomarker expressed in the leukemic but not the normal CD34^+^/CD38^−^ HSC compartment [251,252]. IL1RAP-CART cells react in the presence of IL1RAP^+^ cell lines or primary CML cells, resulting in secretion of proinflammatory cytokines and specifically killing cancer cells *in vitro* and in murine cancer xenograft models. Importantly, IL1RAP-CART cells exhibit cytotoxicity against leukemic stem cells without an apparent effect on CD34^+^ stem cells [253]. Nevertheless, the treatment of myeloid malignancies with CART is challenging because it requires surface antigens that are genuinely expressed by the neoplastic cells [240].

CART cell approaches are presently aimed at targeting leukemic blasts. Due to the inherent capacity of MDSCs and MSCs to inhibit T-cell activation and function, the combined targeting of MDSCs’ or MSCs’ immunosuppressive activities with CART cell immunotherapies appears to be a promising strategy to further improve current therapies for myeloid malignancies. MDSC depletion by the immunotoxin gemtuzumab ozogamicin provides a translational strategy to improve T-cell and CART cell responses against several cancers [254,255]. Interestingly, genetic modification of MSCs to over-express IL-7 and IL-12 may shift the chronic inflammatory profile in the tumor microenvironment to favorable Th1/Th17 for an acute CART cell response by improving and amplifying the antitumor CART cell response [256].

Myeloid malignancies are characterized by abnormalities in both hematopoietic cells and the microenvironment; transformed myeloid cells alter the hematopoietic cell microenvironment function by a direct cell–cell contact and by secretion of proinflammatory and inflammatory cytokines, thus creating a conductive microenvironment for the growth of neoplastic cells. As highlighted in the present review, MDSCs and MSCs are reactive cells that may promote the expansion and protection of tumoral myeloid cells and may reduce the efficacy of chemotherapeutic agents and immunotherapy strategies. Furthermore, MDSCs and MSCs may be used as potential biomarkers for predicting the patient response to treatment or recurrence following therapy and can be useful co-targets in combined current therapies and immunotherapies. Understanding the individual and mutual contribution of MDSCs and MSCs in myeloid malignancies can provide new platforms for developing better and personalized therapies for the treatment of myeloid malignancies and thus improve patients’ quality of life.

## Data Availability

Not applicable.
